# Experimental control of *Triatoma infestans* in poor rural villages of Bolivia through community participation

**DOI:** 10.1093/trstmh/tru205

**Published:** 2015-01-19

**Authors:** Frédéric Lardeux, Stéphanie Depickère, Claudia Aliaga, Tamara Chavez, Lilian Zambrana

**Affiliations:** a Institut de Recherche pour le Développement (IRD), Centre de Bolivie, C.P. 9214, Calle Hernando Siles no. 5290, esq. calle 7, Obrajes, La Paz, Bolivia; b Instituto Nacional de Laboratorios de Salud (INLASA), Laboratorio de Entomología Medica, Calle Rafael Zubieta no. 1889 (lado estado mayor), Miraflores, La Paz, Bolivia; c Universidad Autónoma Gabriel René Moreno, Facultad de Humanidades, Carreras de Comunicación Social y Ciencias de la Educación, Avenida Busch, Santa Cruz de La Sierra, Bolivia

**Keywords:** Bolivia, Chagas disease, Community participation, Risk factor, *Triatoma infestans*, Vector control

## Abstract

**Background:**

*Triatoma infestans* is the main vector of Chagas disease in the southern cone countries. Present control strategies based on indoor and outdoor residual insecticide spraying are not sufficient to control disease transmission, particularly in Bolivia. Techniques based on the management of the human environment may be good alternatives or supplements.

**Methods:**

Social and entomological surveys were carried out in four villages of Bolivia situated in the dry inter-Andean Valleys and the Chaco region. Risk factors for house infestation by *T. infestans* were identified, and an eco-health intervention based on education and community participation was carried out to reduce the risks of house infestation. It consisted of implementing simple and low cost vector control techniques such as coating of mud walls, cleaning activities and removal of poultry that enter rooms to lay eggs.

**Results:**

The eco-health intervention significantly reduced the number of infested bedrooms, the mean abundance of *T. infestans* in bedrooms and beds, especially in the Chaco region. Mud wall coating was well accepted and could be proposed as a supplementary tool to the National Program of Chagas Disease Control to enhance the effects of insecticide sprayings.

**Conclusions:**

Even if cleaning activities were still neglected, community participation proved to be effective in reducing house infestation.

## Introduction

*Trypanosoma cruzi* (Kinetoplastida, Trypanosomatidae), the causal agent of Chagas disease, is transmitted to humans through various routes of which vectorial transmission is predominant and responsible for over 80% of cases.^1^ The control of the disease is therefore strongly dependent on vector control.^2^ The vectors are blood-sucking bugs of the Triatominae family. In all the countries of the ‘southern cone’ *Triatoma infestans* (Hemiptera, Reduviidae) is the main vector and in Bolivia it accounts for more than 98% of bug captures in human dwellings.^3^ This insect entirely completes its life cycle in intra- and peri-domestic habitats, and vector control strategies are therefore based on indoor and outdoor spraying of residual insecticides. The disease is commonly associated with poor rural dwellings at an altitude of between 300 and 3500 m, which represents about 60% of Bolivian territory and involves around 4 million people at risk (approximately 50% of the total population).^4^ Vector control is managed by the National Program for Chagas Disease Control (NPCDC) with the goal of reducing the proportion of infested houses to less than 3%.

Despite some good results obtained with insecticide spraying in the southern cone countries,^5^ Chagas disease remains endemic in large areas of Bolivia and Northern Argentina.^6^ The maintenance of active transmission in villages is mainly due to the reinvasion of human dwellings by residual triatomine populations from areas not well treated in the domicile or in the peridomicile, or from neighboring untreated areas.^7–9^ This problem is enhanced by the recent emergence of foci of resistance to pyrethroid insecticides.^10,11^The present insecticide-based control strategy is therefore not sufficient because it cannot integrate all the factors involved in transmission dynamics.^12^ Limited scale experiments have proven that strategies based on environmental management might be good alternatives or supplements to vector control.^13–18^ Nevertheless, without a better knowledge of environmental factors (ecological and social), these alternatives cannot be optimally implemented at community levels. More insights relevant to specific ecosystems are therefore needed to adequately develop such control techniques into an integrated new strategy.^19^

The present study is focused on an ecosystem approach for the control of Chagas disease in Bolivia by investigating ecological, biological and social (‘eco-bio-social’) factors that may contribute to a more sustainable approach towards vector control. The study was developed in two phases. The first phase was a situational analysis of the eco-bio-social determinants of Chagas disease transmission aimed at identifying risk factors for domicile infestation. In the second phase, integrated vector control techniques based on the management of the domicile environment and aimed at reducing house infestation were implemented through community participation.

## Materials and methods

### Study sites and study design

The study was carried out in the two main endemic regions for Chagas disease, i.e., the dry inter-Andean valleys and the Chaco region. In the dry inter-Andean valleys, situated in the center of Bolivia at an altitude of between 500 and 3300 m, the mean annual temperature ranges from 7–24°C. Mean annual precipitations vary from 500 to 700 mm. In this area, people are mostly of Quechuan descent. The Chaco region is basically a plain with small hills at the south-east of the country. The mean altitude ranges from 200 to 600 m, the mean annual temperature is 25–26°C and precipitations vary from 400 to 900 mm. People are mainly of the Guarani ethnic group. In both regions, small villages are inhabited by subsistence farmers facing hard living conditions.

Potential study villages were identified using the following sampling constraints: households should be grouped to facilitate the social studies such as focus groups and meetings; village size should be of approximately 100 houses to enable complete entomological coverage; villages should be accessible all year long; and villages should have a high house infestation level of *T. infestans* to enable statistical computations. Because regions were homogeneous from a socio-economic perspective, these criteria allowed the selection of similar villages in each region. In each ecological region, two villages were randomly selected from the list of potential villages, of which one was randomly allocated to be the control village, the other one being therefore the intervention village.

The key dependent variables of the study were the presence and abundance of *T. infestans* in the domicile, which were taken as a proxy for Chagas disease transmission.^20, 21^ They were first estimated at baseline during the situational analysis, and then re-assessed after the eco-health intervention. In intervention and control villages, the NPCDC continued its vector control activities once a year, more than four months before the eco-health intervention, with insecticide spraying focused on infested dwellings (alpha cypermethrin at 50 mg active ingredient/m^2^, in the domicile and the peri-domicile area).

In the dry inter-Andean valleys, Eje Pampa (lat -18.54°, long -65.17°, alt 1600 m) was chosen as the intervention village (valley intervention village [VIV]), and Lagar Pampa (lat -18.45°, long -64.99°, alt 1580 m) as the control village (valley control village [VCV]). In the Chaco region, Palmarito (lat -19.49°, long -63.46, alt 940 m) and La Brecha (lat -19.51°, long -62.56°, alt 420 m) were the intervention (Chaco intervention village [CIV]) and the control (Chaco control village [CCV]) villages, respectively.

### Social and entomological surveys

For both social and entomological data, a complete survey was carried out before interventions (baseline data), and again 6 months after interventions using the same protocols.

#### Social surveys

Social surveys consisted of house-to-house surveys with a questionnaire aimed at better understanding knowledge, attitudes and practices (KAP) regarding the vector and Chagas disease; general meetings to discuss the topic; and focus groups. The combination of answers from the questionnaire permitted the design of indices reflecting the knowledge and attitudes towards vector and disease. One point was assigned for each correct answer and 0 otherwise and the total was reduced to a maximum of 1. Each index ranged therefore from 0 to 1 and were used to compare the situations before and after the intervention.

#### Entomological surveys

Using standardized codes, each domestic structure was described in terms of the type of construction material of walls and roof, the wall condition (presence of cracks, crevices and holes), the presence of domestic animals in the room (or at least the presence of nests), the count of frames and posters hanging on walls, the count of bags (50 litres capacity), clothes piles (or similar), cabinets (including large wooden boxes) stuck to the walls; the count of hooked goods (goods hooked on the wall such as clothes, plastic bags etc.); and the count of beds. Triatomines were captured in the domicile by means of active searches by two trained technicians during 60–90 mins per dwelling. No bug irritating product was used.

### Implementation of intervention

The intervention was based on the results of the situational analysis which identified risk factors for house infestation (see Results section). Proposed vector control techniques aimed at reducing vector presence/abundance, respecting the local lifestyle and that could be self-developed by the community without additional cost, were therefore coating walls with a standardized mud mixture (clay, water, straw, animal dung and cactus juice in standardized proportions) that was prepared with products found on site in each village, and used to fill cracks in the walls^22^; house cleaning activities, in particular a thorough cleaning of beds and of objects stuck to the walls; and the removal of animals that enter houses, especially poultry that enter to lay eggs.

The delivery strategy for community participation was identified from the social results and was based on interpersonal communication, community mobilization, lobbying at the community leader level, and supported advertising. The following activities were carried out by the research team: a house-to-house diagnostic of the domestic structures to explain what should be done in each particular situation; a strong social intervention, including school teachers, the participation of community leaders, focus groups, meetings and house-to-house visits. Explanations were given to every householder on the correct way to improve their houses with a correct mud wall coating; a technician could help the householder at the beginning if needed, then the householder had to carry on. The intervention phase lasted 6 months and advances were supervised by the research team.

### Data analysis

The description of the situational analysis was carried out by regression analysis which identified significant risk factors for domestic infestation by *T. infestans*. The numbers of rooms analyzed were 121, 290, 181 and 113 in Palmarito, La Brecha, Eje Pampa and Lagar Pampa, respectively. The presence or absence of *T. infestans* in the domicile was explored by means of logistic regression analysis from which odds ratios were computed. The abundance of *T. infestans* in rooms was explored by means of negative binomial regressions to identify significant non multicolinear environmental variables amongst: ‘number of hooked goods’, ‘number of frames’, ‘number of bags’, ‘number of cloth piles leaning on the walls’, ‘number of poultry nests’, ‘number of beds in room’, ‘room type’ (kitchen, bedroom, store room) and ‘wall condition’ (good, almost good, bad). Probability values of the regression coefficient χ^2^ statistics were used to rank the effects. For the comparison of situations before and after the implementation of the intervention, the presence and abundance of *T. infestans* were computed taking into account only the houses assessed on both occasions. Sources of variations were categorized into the following: the village; the domestic structures (bedroom, kitchens, store-rooms); the locations where *T. infestans* were captured (such as beds; frames; storage bags; piles of items); and wall condition (bad, i.e., with numerous cracks and crevices; fairly good; or very good, i.e., with very few possibilities for *T. infestans* to shelter). The χ^2^ tests were used to compare proportions before and after intervention, and ANOVA (F-tests) were used to compare the evolution of the number of bugs among villages (intervention vs control villages) and time (before vs after intervention). Statistica software version 8 (Statsoft, Maisons-Alfort, France) and PASW version 18 (IBM, Armonk, NY, USA) were used for statistical analysis.

## Results

### Social, cultural, economic and community context

#### House description

In the Chaco region, typical dwellings are generally made of several small mud constructions (20–30m²). Some are used for sleeping (a bedroom has typically 2–3 beds) and others for storing material (store-rooms). They are built by the inhabitants who spread out the mud with their hands over a wood reinforcement made of braided branches (see Supplementary Figure 1). In such walls there are many cracks and crevices which are very favorable sites for the presence of triatomines. The roof is generally made of sheet-iron or thatch. No fences separate neighbors and all the human-made structures are easily accessible for domestic animals (dogs, pigs, goats, cats and chickens) which wander among the constructions and may rest close or even enter into them to sleep.

In the Valleys region, typical dwellings are generally made of one or two constructions having several rooms (bedrooms, store-rooms and kitchens). They are made of mud bricks and walls offer less shelters to triatomines than constructions of the Chaco region (see Supplementary Figure 2). Neighboring houses are separated by walls made of mud bricks. In the backyards of houses, there are small structures and piles of material such as wood and brick that constitute the peri-domicile area in which domestic animals encounter shelter. Most of the families own also a small enclosure (made of a rock base and tree-trunk fence) where goats sleep every night. These structures are generally situated close to houses and may harbor large triatomine colonies.

#### Socio-cultural context

In both regions, the rural population preserve their traditions and cultural identity (Quechua in the Valleys region and Guarani in the Chaco region). Households face economic difficulties, without the possibility of having a paid local job. Most of them are farmers. They sell hardly any products outside their villages (almost none in the Chaco region). As a consequence, men frequently have to work outside their villages and are employed seasonally by large agricultural companies located far away. In all the studied villages, reunions and focus groups showed that the KAP on the vector, Chagas disease and its prevention were deficient. From the KAP survey, it appeared that *T. infestans* can be correctly identified (94.7% [270/285] of correct responses) and can frighten the householder (82.1% [234/285]) because the insect can transmit diseases. Indeed, 87.4% (249/285) of people answered that it can be a disease vector, of which 98.0% (244/249) cited Chagas as the transmitted disease. However, details on the bug's ecology and the disease were still not known: only 30.9% (88/285) of people recognized *T. infestans* eggs; 96.8% (276/285) did not have an idea of the life cycle and longevity of the insect; 28.3% (80/283) did not know that *T. infestans* feeds on blood; 57.5% (164/285) did not know that *T. infestans* is also attracted by domestic animals; 31.9% (91/ 285) of people did not do anything to control the insects; 51.6% (147/285) did not know how Chagas disease can be contracted; only 55.1% (157/285) knew that Chagas disease can be fatal, and of the 40.4% (115/285) of people who knew a patient in their family, 14.8% (17/115) did nothing, although medical consultations at their local health centers are free. In group discussions it was pointed out that house cleaning was not well done and wall coating was neglected.

### Baseline risk factors of house infestation

In the Chaco region, 1465 triatomines were captured in Palmarito (CIV) and 1963 in La Brecha (CCV), which represent an overall mean of 7–12 *T. infestans* per domestic structure. This result contrasted with about one triatomine per structure in the Valleys (in total, 185 insects in Eje Pampa [VIV] and 137 in Lagar Pampa [VCV]).Differences existed according to the use of structures, bedrooms being more infested, in particular in the Chaco region where a mean of about 10–13 *T. infestans* were found (Table 1). Beds were good refuge sites for *T. infestans*, in particular in the Chaco region where a mean of approximately three *T. infestans* and a maximum of 159 insects were found in beds (Table 1). In Palmarito, 47% of the beds were positive for *T. infestans* and 46% in La Brecha. The mean number of *T. infestans* per positive bed rose to 5.0 (SD 10.3) and 6.4 (SD 12.8) in Palmarito and La Brecha, respectively. In the Valleys region <1% of the beds were positive.

**Table 1.** Mean number of *Triatoma infestans* captured in each habitat type, with SD (in parenthesis), and number of structures sampled in each of the four studied villages before interventions

Habitat type	Valleys region		Chaco region	
	Eje Pampa (VIV)	Lagar Pampa (VCV)	Palmarito (CIV)	La Brecha (CCV)
Bedrooms (excluding insects in beds)	1.6 (7.3)	1.9 (6.9)	7.1 (9.6)	3.4 (10.4)
	77	40	105	189
Beds only	0.009 (0.07)	0.8 (2.4)	2.4 (11.7)	2.9 (12.9)
	112	47	277	407
Bedrooms (including insects in beds)	1.6 (7.3)	2.9 (9.7)	12.8 (18.2)	9.5 (20.4)
	77	40	105	189
Store-rooms	0.7 (2.5)	0.3 (1.3)	9.7 (13.1)	2.6 (7.5)
	85	64	12	59
Kitchens	0 (0)	0 (0)	0.2 (0.5)	0.3 (0.9)
	19	9	4	42
Overall	1.0 (5.1)	1.2 (5.9)	12.1 (17.5)	6.8 (17.2)
	181	113	121	290

CCV: Chaco control village; CIV: Chaco intervention village; VCV: Valleys control village; VIV: Valleys intervention village.

The qualitative analysis with logistic regressions (i.e., significant odds ratios) and the quantitative analysis with negative binomial regressions gave results with similar trends. Depending on the studied village, one or more of the following risk factors were identified: bad wall conditions, i.e., when the walls presented cracks and crevices; the presence of bags stuck to the walls; the presence of cloth piles stuck to the wall; the presence of hooked goods on walls; the presence of frames on walls; the presence of poultry nests in the rooms; and the presence of beds in the rooms (Tables 2 and 3). These significant factors can be grouped into two broad categories: living conditions, which encompass wall conditions and cleanliness of the room (beds and various goods stuck to the walls); and the presence of domestic animals in rooms, in particular poultry. In all the villages, the type of room was significant, and bedrooms were the most infested. The wall condition was also significant in all villages; rooms with cracks and holes (wall condition ‘bad’ or ‘almost good’) being the most infested ones. Even if not all risk factors were present in all the villages, the intervention phase was designed as a whole, integrating all the factors in a same global vector control strategy (see Materials and methods).

**Table 2.** The p-values of χ^2^ statistics of the negative binomial regression coefficients

Risk factor	Overall (all 4 villages)	Valleys region			Chaco region		
		Eje Pampa (VIV)	Lagar Pampa (VCV)	Overall	Palmarito (CIV)	La Brecha (CCV)	Overall
Room type^a^	0.00	0.00	0.00	0.00	0.39	0.00	0.00
Bedroom	0.00 (+)	0.00 (+)	0.00 (+)	0.00 (+)	0.76 (+)	0.21 (+)	0.06 (+)
Kitchen	0.00 (−)	0.03 (−)	1.00 (−)	0.01 (−)	0.28 (−)	0.00 (−)	0.00 (−)
Wall condition^b^	0.00	0.04	0.00	0.00	0.00	0.00	0.00
Almost good	0.00 (+)	0.07 (+)	0.00 (+)	0.03 (+)	0.02 (+)	0.00 (+)	0.00 (+)
Bad	0.00 (+)	0.01 (+)		0.00 (+)	0.00 (+)	0.00 (+)	0.00 (+)
Hooked goods	0.03	0.05	0.28	0.70	0.91	0.31	0.68
Frames	0.07	0.02	0.03	0.00	0.86	0.18	0.61
Bags	0.14	0.04	0.25	0.00	0.93	0.40	0.49
Cloth piles	0.00	0.97		0.43	0.05	0.32	0.00
Poultry nests	0.00	0.00	0.77	0.01	0.00	0.00	0.00
Beds	0.00	0.01	0.93	0.00	0.04	0.00	0.00

CCV: Chaco control village; CIV: Chaco intervention village; VCV: Valleys control village; VIV: Valleys intervention village.

In parenthesis is the sign (+) or (-) of the *b* coefficient of the regression, indicating the direction of the correlation in relation to the reference category: wall condition in La Brecha is significant, and when wall condition is ‘almost good’ or ‘bad’, there are more *Triatoma infestans* in the room than when the wall condition is ‘very good’. Similarly, the room type is significant, and there are less *T. infestans* in kitchen than in store-rooms.

^a^ ‘store-room’ is the reference category.

^b^ ‘good’ is the reference category.

**Table 3.** Odds ratios for risk factors and their 95% CIs (in parenthesis) from logistic regressions of the presence of *Triatoma infestans* in domestic structures

Risk factor	Overall (4 villages)	Valleys region			Chaco region		
		Eje Pampa (VIV)	Lagar Pampa (VCV)	Overall	Palmarito (CIV)	La Brecha (CCV)	Overall
Bad wall condition^a^	0.42 (0.27–0.65)	1.96 (0.40–9.76)	NC	4.14 (0.94–18.21)	4.28 (1.59–11.47)	3.34 (1.57–7.09)	3.92 (2.29–6.70)
Bad roof condition^b^	1.26 (0.80–1.98)	1.50 (0.16–13.62)	0.68 (0.07–6.83)	0.73 (0.19–2.83)	1.03 (0.33–3.27)	3.74 (1.61–8.71)	0.64 (0.36–1.14)
Presence of hooked goods	0.98 (0.70–1.39)	0.74 (0.29–1.90)	1.05 (0.34–3.25)	1.00 (0.51–1.99)	1.85 (0.65–5.22)	0.99 (0.55–1.78)	1.19 (0.73–1.92)
Presence of frames	0.97 (0.66–1.42)	0.80 (0.25–2.63)	2.01 (0.56–7.17)	1.39 (0.63–3.05)	1.81 (0.52–6.35)	0.89 (0.46–1.70)	1.18 (0.68–2.02)
Presence of bags	1.99 (1.20–3.29)	3.01 (1.15–10.5)	3.36 (0.68–16.58)	2.98 (1.19–7.49)	0.63 (0.11–3.46)	2.13 (1.09–4.61)	1.45 (0.73–2.89)
Presence of cloth piles	1.57 (1.02–2.41)	0.65 (0.13–3.22)	––	0.55 (0.11–2.62)	1.07 (0.25–4.52)	0.88 (0.46–1.67)	0.73 (0.42–1.27)
Presence of poultry nests	1.98 (1.30–3.07)	1.97 (0.78–4.94)	2.29 (0.69–7.60)	2.05 (1.03–4.07)	10.45 (1.51–72.28)	4.54 (1.63–12.65)	6.11 (2.61–14.29)
Presence of beds in room	6.87 (4.63–10.19)	1.31 (0.48–3.53)	1.25 (0.38–4.15)	1.13 (0.55–2.35)	6.18 (1.45–26.26)	12.97 (6.46–26.02)	11.73 (6.49–21.17)

CCV: Chaco control village; CIV: Chaco intervention village; NC: not estimated; VCV: Valleys control village; VIV: Valleys intervention village.

^a^ A wall was estimated in bad condition when numerous cracks and crevices were observed, permitting the survival of *T. infestans*.

^b^ A roof was estimated in bad condition when it was made of thatch.

#### Eco-health intervention: community participation and impact on house infestation

Because kitchens were not numerous and had low infestation rates, and because store-rooms were also less frequently infested (Table 1), results comparing the ‘before and after’ situation focus only on bedrooms.

#### Social context

At the beginning of the study, 96% (44/47) and 100% (92/92) of the families were willing to participate in the study in Eje Pampa (VIV) and Palmarito (CIV) respectively, and 96% and 82% did participate effectively. Indeed, the percentage of treated bedrooms was significantly higher in the intervention villages (60–70%) than in the control villages (<10%, that represents in fact the usual maintenance of houses) (Table 4). KAP indices indicated that knowledge and practices related to the vector and disease significantly improved after the intervention in Eje Pampa and Palmarito (Table 5). After intervention in the intervention villages, the belief that it was possible to eliminate *T. infestans* from houses rose from 37% (34/92) to 90% (66/73) in Palmarito ($\chi_{1}^{2}=46$; p=0) and from 79% (37/47) to 90% (36/40) in Eje Pampa ($\chi_{1}^{2}=1.23$; p=0.25). The usefulness of cleaning activities for lowering bug densities was better understood in the intervention villages than in the control villages. Indeed, in Eje Pampa (VIV) and Palmarito (CIV), 45% (18/40) and 60% (44/73) of the respective families thought that cleaning activities reduced *T. infestans* abundance in their homes, versus only 9% (3/34) in Lagar Pampa (VCV) and 18% (16/91) in La Brecha (CCV). However, the management of domestic animals was not well understood. The social survey carried out 6 months after intervention pointed out that in all the four villages, 80% of people declared that hens still entered their houses to lay eggs. Indeed, the proportion of bedrooms with visible poultry nests did not vary significantly before and after the intervention, decreasing from 21 to 13% (p=0.14) in Eje Pampa (VIV) and from 16 to 9% (p=0.07) in Palmarito (CIV). Similarly, a non-significant decrease of the number of visible nests in bedrooms was generally observed in the villages: 37% in Eje Pampa (VIV; initial nest number 3; p=0.08), 13% in Lagar Pampa (VIV; initial nest number 43; p=0.27) and 13% in Palmarito (CIV; initial nest number 33; p=0.14). On the contrary, in La Brecha (CCV), there was a significant increase of 35% of poultry nests (p=0.04) which rose from 31 to 42.

**Table 4.** Percentage of bedrooms in which wall condition was bad, fairly good or good; proportion of *Triatoma infestans*-positive bedrooms, and overall mean number of bugs captured (excluding beds)

	Before intervention					After intervention					
	Wall condition (%)			% positive bedrooms	Mean bug abundance (SD), n	Wall condition (%)			% positive bedrooms	% bedrooms intervened^a^	Mean bug abundance (SD) n
Village	Bad	Fairly good	Good			Bad	Fairly good	Good			
Palmarito (CIV)	75.8% (276/364)	12.1% (44/364)	12.1% (44/364)	72.5% (66/91)	6.9 (9.5), 91	33.9% (137/404)	15.1% (61/404)	50.0% (202/404)	20.8% (21/101)	65.3% (66/101)	0.6 (1.9), 101
La Brecha (CCV)	57.1% (354/620)	21.9% (130/620)	21.9% (136/620)	50.9% (79/155)	3.6 (11.3), 155	55.0% (352/640)	20.0% (128/640)	25.0% (160/640)	36.2% (58/160)	5.0% (8/160)	2.4 (9.3), 160
Eje Pampa (VIV)	85.4% (164/192)	9.9% (19/192)	4.7% (9/192)	14.6% (7/48)	0.6 (2.0), 48	24.5% (54/220)	37.7% (83/220)	37.7% (83/220)	9.1% (5/55)	72.7% (40/55)	0.4 (1.6), 55
Lagar Pampa (VCV)	56.2% (72/128)	31.2% (40/128)	12.5% (16/128)	21.9% (7/32)	2.3 (7.7), 32	62.1% (92/148)	18.9% (28/148)	18.9% (28/148)	13.5% (5/37)	5.4% (2/37)	1.1 (5.6), 37

CCV: Chaco control village; CIV: Chaco intervention village; VCV: Valleys control village; VIV: Valleys intervention village.

^a^ The proportion of bedrooms in which owners have mud-coated the walls as compared to the baseline condition.

**Table 5.** Knowledge, attitudes and practices (KAP) indices on vector and disease knowledge and disease attitude before and after intervention in the intervention villages (maximum value for each index: 1, indicating a 100% of correct responses to questions). The $\chi_{1}^{2}$ test compares the indices before and after intervention in each village

KAP index	Eje pampa (Valley intervention village)			Palmarito (Chaco intervention village)		
	Before intervention	After intervention	$\chi_{1}^{2}$	Before intervention	After intervention	$\chi_{1}^{2}$
Vector knowledge	0.86	0.97	6.4 (p=0.01)	0.76	0.93	9.7 (p=0)
Vector attitude	0.87	0.99	9.3 (p=0)	0.91	0.98	3.4 (p=0.03)
Disease knowledge	0.73	0.97	20.7 (p=0)	0.53	0.96	46.4 (p=0)

#### House infestation

The mean number of captured *T. infestans* in bedrooms decreased significantly in the intervention villages, in particular in the Chaco region, where values were extremely high at the beginning of the study (Table 4). ANOVA computations with the villages and the ‘before and after’ situation as the two factors indicated that there was no difference in the mean number of *T. infestans* among villages in the Chaco region at baseline (*F*=1.05; p=0.30), but was significantly lower in the intervention village after intervention (*F*=17.7; p=0). The proportion of bedrooms positive for *T. infestans* followed the same downward trend before and after interventions, in particular in the Chaco region (Table 4).The percentage of positive bedrooms decreased approximately 70% in Palmarito (CIV) and only approximately 30% in La Brecha (CCV). The decrease was significantly greater in the intervention village than in the control village ($\chi_{1}^{2}$=7.68; p<0.05). In the Valley region, the same trend was observed but the differences were not significant (approximately 40% reduction in both villages). Beds were also impacted as highlighted by the significant decrease of the proportion of positive beds and the mean number of bugs per bed before and after intervention (Table 6). In Palmarito (CIV), the proportion of positive beds decreased from 47% to 12% (significant decrease of 74% [$\chi_{1}^{2}=6.56$; p<0.05]), and the mean number of *T. infestans* also decreased significantly from 4.7 to 1.2 (*F*=13.8; p=0) whereas in La Brecha, although the proportion of positive beds significantly decreased after intervention ($\chi_{1}^{2}=30$; p=0), the mean number of *T. infestans* per bed increased (Table 6). In the Chaco region, the number of *T. infestans* captured behind objects stuck to walls decreased significantly in the intervention village, dropping from 91 to 18, while in the control village values were 99 before intervention and 157 after intervention. This decrease in the intervention village is likely due to the combined effects of wall mud-coating and cleaning activities. In the valley villages, numbers of captured *T. infestans* were too low (0 in Eje Pampa) to enable such comparisons. Even if there was a significant decrease in the mean number of *T. infestans* in the control villages, likely due to natural fluctuations of vector populations, or to the effects of insecticide spraying by NPCDC or the activities of the research group (e.g., by removing bugs from structures and beds), the decline was more important in the intervention villages, indicating that the impact of control actions was substantial.

**Table 6.** Number of beds sampled, proportion of beds positive for *T. infestans,* mean number of bugs per bed and standard deviation before and after the eco-health intervention

Village	Before intervention				After intervention			
	No. beds sampled	Positive beds (n)	Mean bug abundance per bed	SD	No. beds sampled	Positive beds (n)	Mean bug abundance per bed	SD
Palmarito (CIV)	235	46.8% (110)	4.7	12.6	240	12.1% (29)	1.2	9.0
La Brecha (CCV)	321	43.9% (141)	3.8	13.6	316	23.1% (73)	5.3	21.7
Eje Pampa (VIV)	74	1.3% (1)	0.01	0.1	78	0% (0)	0	NA
Lagar Pampa (VCV)	38	2.6% (1)	0.4	2.8	46	0% (0)	0	NA

CCV: Chaco control village; CIV: Chaco intervention village; NA: Not applicable; VCV: Valley control village; VIV: Valley intervention village.

## Discussion

In the studied villages the baseline survey indicated that a very large percentage of householders knew that *T. infestans* is the Chagas disease vector. However, people did not have good practices to avoid the presence of the insect in their domicile which were therefore highly infested. The eco-health approach used in the present study demonstrates that basic techniques implemented through community participation (mud wall coating [see Supplementary Figures 3 and 4 showing the ‘before’ and ‘after’ coating of houses], cleaning activities and the removing of poultry from homes) may significantly decrease *T. infestans* densities in the domiciles.

Most people worked at least a bit in mud wall coating, even if the work has not always been perfect. Indeed, the proportion of bedrooms intervened increased significantly in the intervention villages where 65% and 76% of bedrooms were finished or almost finished in Palmarito and Eje Pampa, respectively. In general, people argued about their lack of availability and lack of workforce to explain their non-compliance. The coating of walls with mud, which respects the local lifestyle for house construction, appears to be a method of choice to strongly decrease the infestation rate. Furthermore, mud coating is a robust construction technique that may last several years.^22^ However, only coating houses (even combined with insecticide spraying) is not sufficient to eliminate *T. infestans*.^23^ Our current data suggest that when it is well done, mud wall-coating has a strong impact on bug densities, and agree with previous studies indicating that if wall coating is partial or poorly done, triatomine populations will not disappear.^14,23^

Cleaning activities aimed at killing *T. infestans* hidden behind objects stuck to the walls has been found efficient.^14^In the present study, the mean number of *T. infestans* captured behind bags, hooked goods, frames and in beds decreased significantly in the intervention villages. However, this could be attributed not only to the cleaning activities but also to a synergic effect of all the control techniques involved, including the research team samplings and the insecticides sprayings of the NPCDC.

The present study identified another risk factor for house infestation: the presence of poultry that lay eggs inside rooms. Although the number of hen nests inside houses decreased in the intervention villages, too many people (111/139, 79.8%) said that poultry still enter their house although they learned (129/139, 92.8%) that domestic animals can attract *T. infestans*. Although knowledge on the role of poultry in house infestation was improved, the ‘good practices’ for animal management were still difficult to establish.

### Limitations

The management of the ecosystem in which domestic vectors develop, with community participation, has long been a promising idea and in some instance has given good results.^24^ However, changing attitudes and practices in poor indigenous communities which have deeply rooted habits and traditions is a long process. Sustainability is a key parameter in community participation. ‘Good practices’ may be acquired, but if not regarded as a priority could be abandoned in the medium term. In Bolivia where poor farmers do not see Chagas disease as a real health problem, the proposed control techniques of the study may vanish with time. For example, the technique of mud coating needs some maintenance and if people cannot connect the technique with vector control and leave cracks that re-appear on walls, then vector control will become less effective. New constructions and newcomers have also to adopt the ‘good practices’. A sufficient number of villagers should therefore be involved in such practices to give examples and encourage newcomers to follow them. Another limitation is related to the neighboring peri-domestic structures such as animal enclosures or abandoned houses that are used as refuges by wandering domestic animals (pigs, goats, dogs) in which insect colonies can develop. They may jeopardize the successful control of intra-domestic populations of *T. infestans* as they might be re-infestation sources.^8,9,25^ This could be a problem in the Valley region where *T. infestans* is found in greater abundance in the peri-domestic area. As shown by the present study, long-term sustainability of the proposed eco-health approach will therefore be dependent on very careful, thorough and frequent vigilance of domestic infestations and will have to take into account additional strategies directed to peri-domestic structures.

### Conclusions

To control vectors that live in association with humans, community participation is a key factor as none of the present control techniques (including insecticide spraying) has proven to be sufficient to control disease transmission in a sustainable fashion in Bolivia. Focus groups pointed out a lack of education in cleaning activities aimed at eliminating *T. infestans* and, therefore, a first step toward sustainability could be the incorporation of basic teaching on cleaning activities (combined with lessons on house construction aimed at vector elimination) in the school programs. For the cleaning of bed structures, one alternative might be the use of insecticide-impregnated bednets which has long been proved to be a potentially cost-effective and sustainable option.^26, 27^ In the short term, the coating of walls with mud could be proposed as a supplementary tool to the NPCDC to enhance the effects of the usual insecticide spraying. Long term sustainability may be achieved with the participation of various stakeholders including local authorities and NPCDC who seem interested in eco-health approaches to control Chagas disease.

## Supplementary data

Supplementary data are available at Transactions Online (http://trstmh.oxfordjournals.org/).

Supplementary Data
