# Supplementary material for: Experimental control of Triatoma infestans in poor rural villages of Bolivia through community participation
Source: Trans R Soc Trop Med Hyg. 2015 Jan 19;109(2):150–8. doi: 10.1093/trstmh/tru205 (PMC4299529; doi:10.1093/trstmh/tru205)
Supplement: Supplementary Data [file supp_109_2_150__index.html]

Supplementary Data 

# Experimental control of *Triatoma infestans* in poor rural villages of Bolivia through community participation

## Supplementary Data

Supplementary Data

**Files in this Data Supplement:**

- Supplementary Figure 1 - tif file
- Supplementary Figure 2 - tif file
- Supplementary Figure 3 - tif file
- Supplementary Figure 4 - tif file
