# Supplementary figures and images for: Experimental control of Triatoma infestans in poor rural villages of Bolivia through community participation
Source: Trans R Soc Trop Med Hyg. 2015 Jan 19;109(2):150–8. doi: 10.1093/trstmh/tru205 (PMC4299529; doi:10.1093/trstmh/tru205)

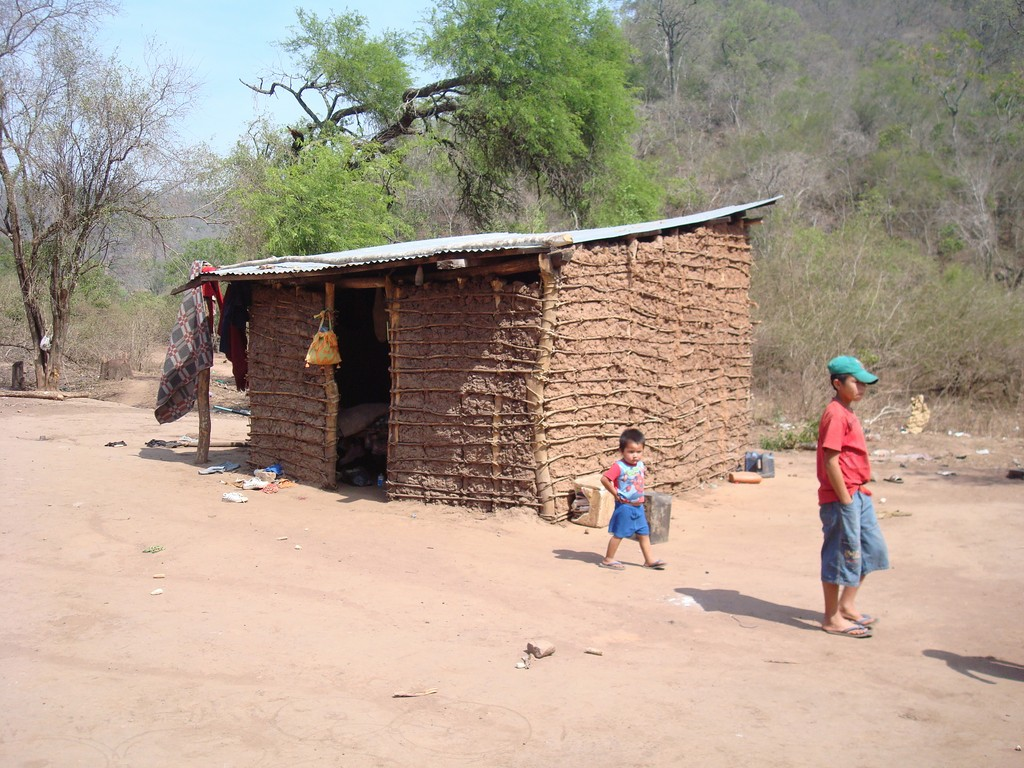

Supplement: Supplementary Data [file supp_tru205_tru205supp_fig1.tif]

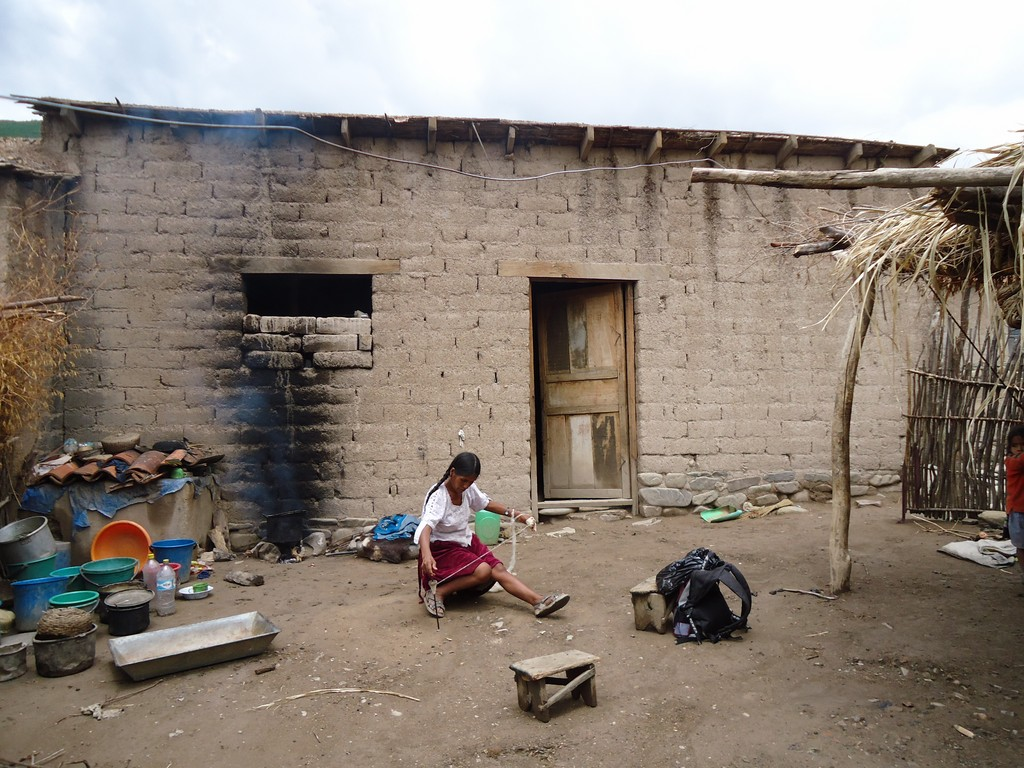

Supplement: Supplementary Data [file supp_tru205_tru205supp_fig2.tif]

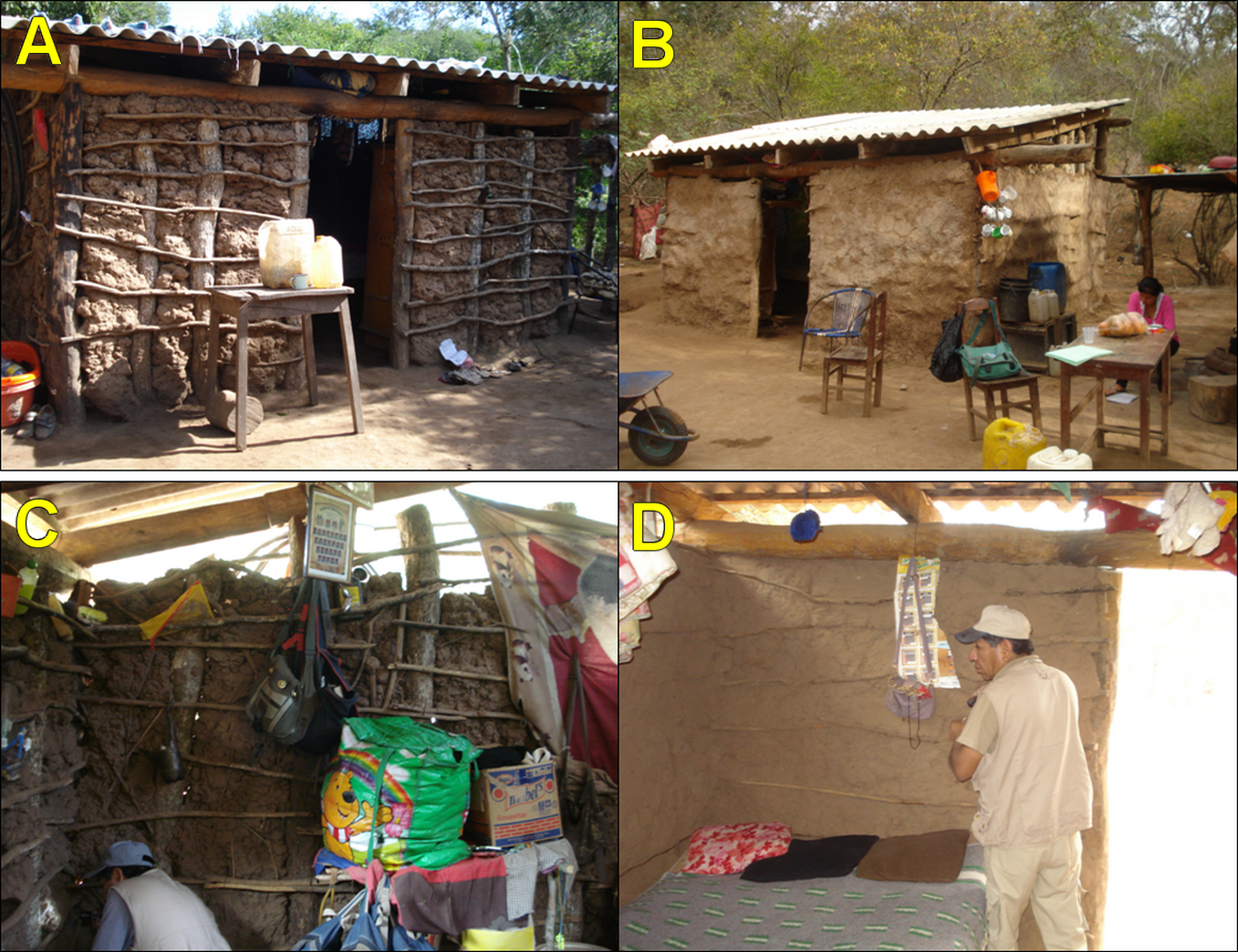

Supplement: Supplementary Data [file supp_tru205_tru205supp_fig3.tif]

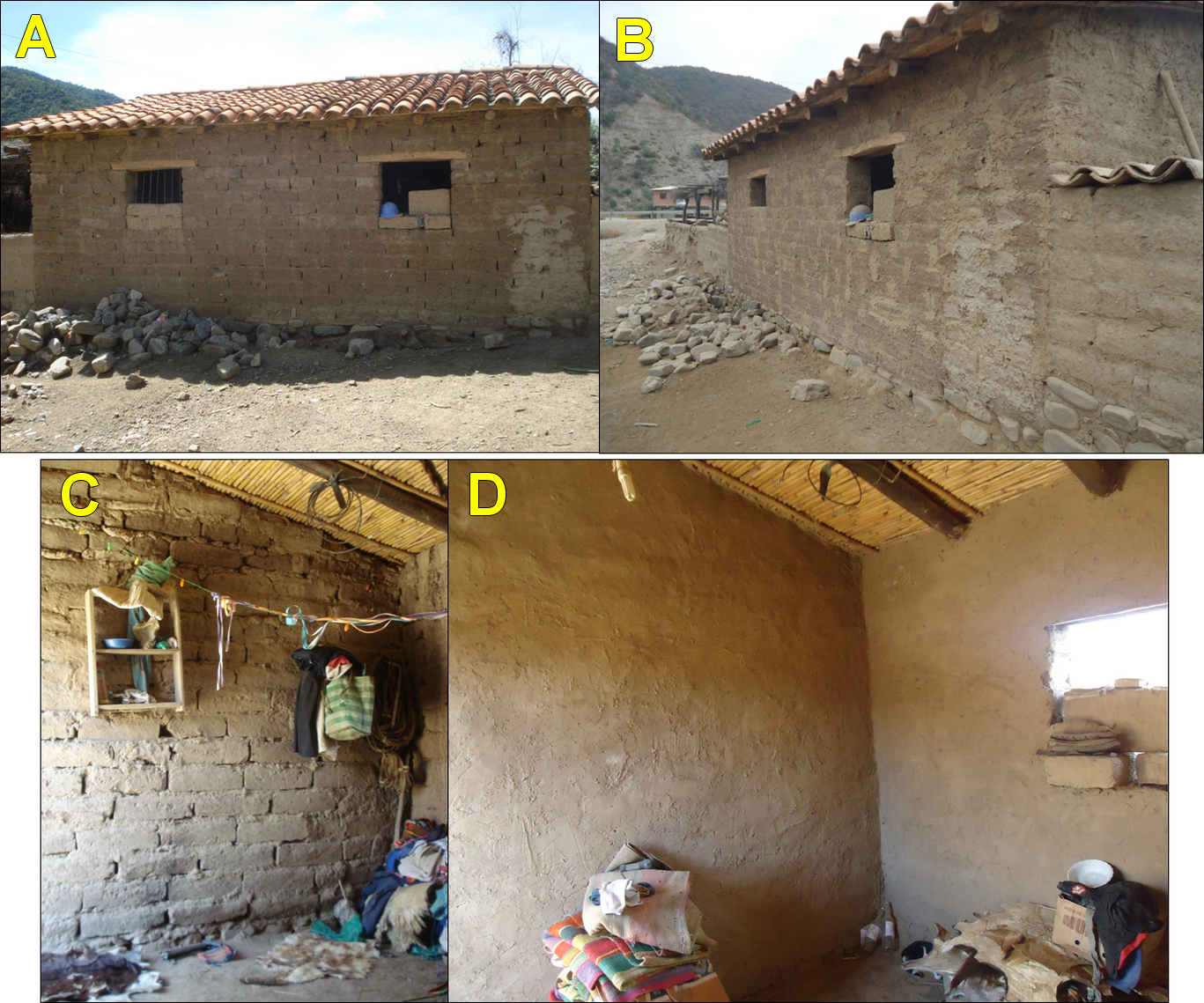

Supplement: Supplementary Data [file supp_tru205_tru205supp_fig4.tif]
